# Heterogeneity of amplification of HER2, EGFR, CCND1 and MYC in gastric cancer

**DOI:** 10.1186/s12876-015-0231-4

**Published:** 2015-02-05

**Authors:** Phillip Stahl, Carsten Seeschaaf, Patrick Lebok, Asad Kutup, Maximillian Bockhorn, Jakob R Izbicki, Carsten Bokemeyer, Ronald Simon, Guido Sauter, Andreas H Marx

**Affiliations:** 1 Institute of Pathology, University Medical Center Hamburg-Eppendorf, Hamburg, Germany; 2 General, Visceral and Thoracic Surgery Department and Clinic, University Medical Center Hamburg-Eppendorf, Hamburg, Germany; 3 II Med. Klinik, Oncology, Hematology with section Pneumology, University Medical Center Hamburg-Eppendorf, Hamburg, Germany

**Keywords:** HER2, EGFR, MYC, CCND1, Gene amplification, Gastric cancer, Heterogeneity tissue microarray

## Abstract

**Background:**

Intra-tumor heterogeneity is a potential cause for failure of targeted therapy in gastric cancer, but the extent of heterogeneity of established (HER2) or potential (EGFR, CCND1) target genes and prognostic gene alterations (MYC) had not been systematically studied.

**Methods:**

To study heterogeneity of these genes in a large patient cohort, a heterogeneity tissue microarray was constructed containing 0.6 mm tissue cores from 9 different areas of the primary gastric cancers of 113 patients and matched lymph node metastases from 61 of these patients. Dual color fluorescence in-situ hybridization was performed to assess amplification of HER2, EGFR, CCND1 and MYC using established thresholds (ratio ≥ 2.0). Her2 immunohistochemistry (IHC) was performed in addition.

**Results:**

Amplification was found in 17.4% of 109 interpretable cases for HER2, 6.4% for EGFR, 17.4% for CCND1, and 24.8% for MYC. HER2 amplification was strongly linked to protein overexpression by IHC in a spot-by-spot analysis (p < 0.0001). Intra-tumor heterogeneity was found in the primary tumors of 9 of 19 (47.3%) cancers with HER2, 8 of 17 (47.0%) cancers with CCND1, 5 of 7 (71.4%) cancers with EGFR, and 23 of 27 (85.2%) cancers with MYC amplification. Amplification heterogeneity was particularly frequent in case of low-level amplification (<10 gene copies). While the amplification status was often different between metastases, unequivocal intra-tumor heterogeneity was not found in individual metastases.

**Conclusion:**

The data of our study demonstrate that heterogeneity is common for biomarkers in gastric cancer. Given that both TMA tissue cores and clinical tumor biopsies analyze only a small fraction of the tumor bulk, it can be concluded that such heterogeneity may potentially limit treatment decisions based on the analysis of a single clinical cancer biopsy.

**Electronic supplementary material:**

The online version of this article (doi:10.1186/s12876-015-0231-4) contains supplementary material, which is available to authorized users.

## Background

Although its incidence and mortality is declining, gastric adenocarcinoma remains the second most frequent cause of cancer death worldwide [1-3]. Similar to most other cancers, prognosis and therapeutic options depend on clinicopathological parameters [4-6]. Surgery is the only curative treatment, which is supplemented by neoadjuvant and adjuvant (radio-) chemotherapy [7,8]. As our knowledge on the molecular biology of gastric cancer increases rapidly, it can be hoped, that the identification of molecular mechanisms driving cancer aggressiveness, influencing response to specific chemotherapies, and representing direct targets for therapeutic drugs will eventually enable a better treatment of our patients.

Gene amplification is a prime mechanism of cancer cells for overexpressing genes that are critical for their survival and expansion. Determination of the amplification status is thus often clinically relevant. For example, MDM2 amplification is a diagnostic hallmark in liposarcoma [9], CCND1 amplification is a suspected predictor of resistance against hormone therapy in breast cancer [10], MYC amplification is a strong prognostic marker in breast cancer and other tumors [11], MYCN amplification is an established prognostic marker in neuroblastoma [12], and HER2/EGFR are established therapeutic targets that are often overexpressed as a consequence of gene amplification [13,14]. On a diagnostic level, gene amplification has the advantage that the amplification level can be precisely measured on a cell by cell basis by fluorescence in situ hybridization (FISH). FISH analysis is unaffected by the (unavoidable) admixture of normal cells to cancer tissues and FISH results are not altered by tissue processing variations.

However, even if molecular methods work at the highest level of precision, diagnostic errors will occur in heterogeneous cancers. For practical purpose, the molecular tumor status is generally determined on a small fraction of the primary tumor, for example derived from a biopsy. Such information will not always reflect the molecular situation of the entire (heterogeneous) cancer mass. Molecular heterogeneity may not only lead to clones with different aggressiveness within one cancer but also to subpopulations with varying response to anti-cancer drugs and thus be of utmost clinical significance. Nevertheless, even in most relevant cancer types and for highly promising diagnostic and therapeutic molecules, intra-tumor genetic heterogeneity is not analyzed in systematic studies to an extent that reflects the importance of the topic.

Thus, the aim of our study was to gain deeper insight in genomic heterogeneity in gastric cancer in a larger scale. For this purpose, we selected 4 genes (HER2, CCND1, MYC and EGFR) with established relevance for gastric cancer, which were previously reported to be amplified. Due to its analytical precision [13], FISH is optimally suited for determining intra-tumor heterogeneity. In contrast to IHC analyses, FISH does not suffer from false-negative findings since non-reactive tissues can easily be identified by the absence of the FISH signals. Reliable analysis results are critical in heterogeneity analysis, because every false measurement would immediately result in false categorization of a tumor as “heterogeneous”. In a potential diagnostic setting, false heterogeneity calling could even lead to the exclusion of patients who could benefit from targeted therapy. To systematically study molecular heterogeneity of gastric cancer we further generated a new tissue microarray (TMA) based platform. In this “heterogeneity TMA” approach, a multitude of tissue cores are sampled per patient both from the primary tumor and various metastases.

## Methods

### Gastric cancer tissues

A total of 113 gastric cancers treated by gastrectomy and lymphadenectomy between 1995 and 2010 at the University Medical Center Hamburg-Eppendorf, and having at least one retained cancer containing tissue block, were included in this study. The use of archived diagnostic left-over tissues and their analysis for research purposes (manufacturing of tissue microarrays) has been approved by the local ethics committee (Ethics commission Hamburg, WF-049/09), and has been carried out in compliance with the Helsinki Declaration. Informed consent has not been collected specifically for the patient samples included in this study. This is in accordance with local laws (HmbKHG, §12,1).

All tissues had been fixed in 4% buffered formalin and were paraffin embedded. The average number of tumor containing blocks was 3.7 (range: 1–9). Lymph node metastases were identified in 61 of these patients. Among these nodal positive patients, 24 had one, 7 had two, 13 had three, and 17 had more than three metastases. The maximal number of metastases was 9. The patient age ranged between 35 to 89 years (median 62 years). The clinical and pathological features of our tumor collection are provided in Additional file 1: Table S1.

### Tissue microarray (TMA) construction

From the 113 primary tumors, 9 different tissue cylinders were taken from tumor containing tissue blocks for TMA manufacturing in order to obtain an optimal representation of the entire tumor mass. Tissue blocks with the highest tumor content were selected for TMA making after careful histological revision of all archived tissue blocks. Tissue blocks with small cancer areas (less than approx. 2 mm^2^) were excluded from the study. If less than 9 eligible tumor containing blocks were available from the primary tumor, multiple punches were taken from one or several tumor blocks. Emphasis was then placed on having a most representative sampling with as equal as possible distances between the selected cores and equal representation of all tumor foci. In addition, a total of 435 tissue cores were taken from the 174 metastases of the 61 patients with metastatic disease, including 9 cores from the metastases of 37 patients, 6 cores from the metastases of 10 patients, and 3 cores from the metastases of 14 patients. On average, there were 2.5 tissue cores (range 1–9) per metastasis, including 3–9 cores per metastasis from the 24 patients with one metastasis, 2–7 cores per metastasis from the 7 patients with 2 metastases, 3 cores per metastasis from the 13 patients with 3 metastases, and 1–9 cores per metastasis from the 17 patients with more than 3 metastases. TMA construction was performed as described before [15]. In brief, hematoxylin and eosin (HE)-stained sections were made from each block to define representative tumor regions. One tissue cylinder with a diameter of 0.6 mm was then punched from each tumor “donor” tissue block using a homemade semi-automated precision instrument and brought into empty recipient paraffin blocks. Four μm sections of the resulting TMA blocks were transferred to an adhesive coated slide system (Instrumedics Inc., Hackensack, NJ). Consecutive sections were used for fluorescence in situ hybridization (FISH) and immunohistochemistry (IHC).

### Fluorescence in situ hybridization

Freshly cut TMA and conventional large sections were used for FISH analysis. A commercial kit (paraffin pretreatment reagent kit 1, Abbott Laboratories, IL, USA) was used for proteolytic slide pretreatment. Four different probe sets were hybridized according to manufacturer’s instructions, including the PathVysion HER2/CEP17 probe set (Abbott 02J01-030) for HER2 copy number detection, LSI EGFR Spectrum Orange/CEP7 Spectrum Green probe set (Abbott Cat. # 05J48-001) for EGFR copy number detection, LSI CCND1 Spectrum Orange/CEP11 Spectrum Green probe set (Abbott Cat. # 3N88-20) for Cyclin D1 copy number detection, and the Zytolight Spec c-Myc/Cen8 dual color probe (ZytoVision Cat. # Z-2092-200) probe set for c-MYC detection. Prior to hybridization, sections were deparaffinized, air-dried, dehydrated and denatured for 5 minutes at 74°C in 70% formamide-2x SSC (tri-sodium citrate sodium chloride) solution. After overnight hybridization at 37°C in a humid chamber, slides were washed and counterstained with 0.2 μmol/L DAPI (4′-6-diamidino-2-phenylindole) in anti-fade solution.

### Scoring of FISH signals

**Figure 1:**
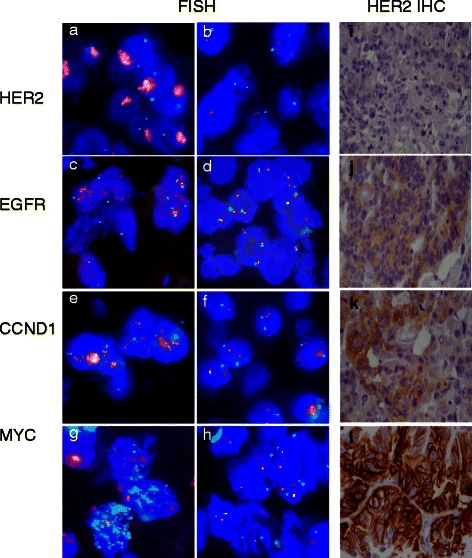
**Representative images of FISH- and IHC findings in gastric cancer. a-h)** Examples of amplified and non-amplified cancers: **a, b)** HER2, **c, d)** EGFR, **e, f)** CCND1 and **g, h)** MYC. **i-l)**: Examples of HER2-immunohistochemistry negative (score 0–1, **i**, **j**) and positive (score 2–3, **k**, **l**) cancers.

Each tumor spot was carefully visually evaluated, and the predominant score, including gene and centromere signal copy numbers as well as the gene-to-centromere ratio, was recorded for each FISH probe in at least 20 non-overlapping cell nuclei. Data from our laboratory have previously shown that diagnosis of amplification based on signal number estimation is highly reliable [16]. A tumor was considered amplified if at least twice as much gene signals as compared to the corresponding centromere signals were observed (ratio gene: centromere ≥2.0). High-level amplification was considered if more then 10 gene signals were counted. All other tumors were considered non-amplified. Figure 1a-h gives examples of amplified and non-amplified tumors.

### HER2 immunohistochemistry

HER2 expression was analyzed using the HercepTest (DAKO) according to the protocol of the manufacturer. Antigen retrieval of the deparaffinized tissue sections was performed in a water bath at 95°C to 99°C for 50 minutes followed by peroxidase blocking and incubation with the prediluted primary antibody. Cell line test slides provided by the manufacturer were used as positive and negative controls. Immunostaining was scored following a 4-step scale (0, 1+, 2+, 3+) according to the consensus panel recommendations on HER-2 scoring for gastric cancer [17]. Scores 0–1 were considered negative, scores 2–3 positive for HER2 expression. Examples of HER2 negative and HER2 positive immunostainings are given in Figure 1i-l.

### Assessment of heterogeneity

Only tumors with at least 3 interpretable tissue spots in the primary cancers were included in heterogeneity analysis. A tumor was considered homogeneous for a given marker (i.e., amplification of HER2, CCND1, EGFR, c-MYC and expression of HER2) if all analyzable tissue spots showed an identical result. All other tumors were considered heterogeneous.

### Large section validation

Three cases with suspicious heterogeneous findings on the TMA (amplification in the metastases without amplification in the primary tumor) were subjected to large section analysis. In these cases, presence of tumor cells was first verified in each spot by comparison of an H & E stained consecutively cut reference section of the TMA to validate heterogeneity. Conventional 4 μm large sections were then cut from the corresponding cancer-containing tissue blocks and subjected to FISH analysis as described before.

### Statistics

Chi^2^ test and contingency tables were used to compare results of HER2 FISH and IHC analyses.

## Results

### Technical issues

For all patients data from primary tumors were only included in our analysis if at least 3 of 9 cores had interpretable data. Data from metastases were always included, even if only one spot was interpretable as long as the corresponding primary cancer was informative with respect to this study. The rate of informative primary tumors (PT) and metastases (M) was 109 (PT) and 61 (M) for each of HER2, CCND1, c-MYC and EGFR FISH, as well as HER2 IHC. Reasons for non-informative results included non-interpretable FISH results due to insufficient hybridization, a lack of unequivocal tumor on certain spots and completely lacking spots on TMA sections.

### HER2 FISH

**Figure 2:**
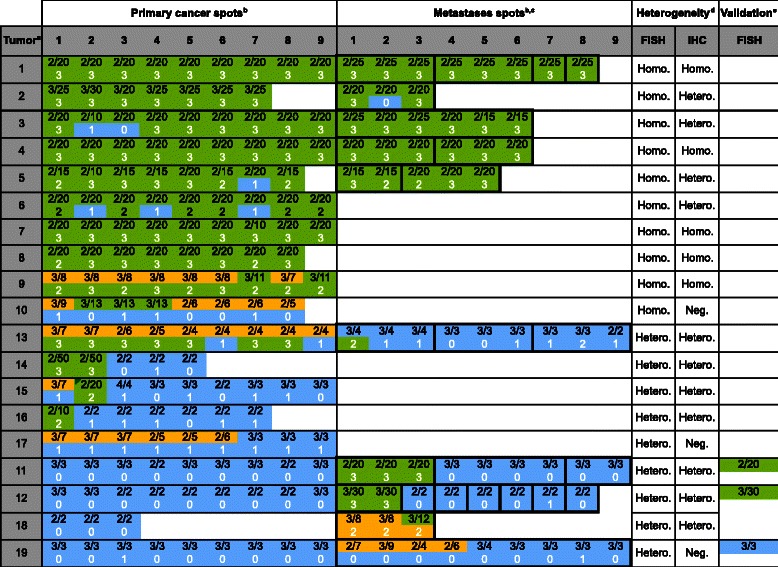
**HER2 FISH and IHC findings in primary gastric cancers and their metastases in 19 cancers with HER2 amplification.**^a^“Tumor” indicates the cases number referred to in the text. ^b^The FISH and IHC results of individual tissue spots are indicated by integers reflecting the copy numbers of centromere 17/HER2 (i.e. 2/20, black face) and the HercepTest IHC score (i.e. 3, white face) in the primary and metastases spots. Green color in the primary/metastases spots area indicates high-level HER2 amplification or positive IHC findings, orange color indicates low-level amplification, and blue color indicates negative IHC findings. ^c^Bold black outline in the “metastases spots” highlights spots that were derived from the same metastasis. ^d^“Heterogeneity” indicates whether FISH or IHC findings were homogeneous (Homo.) or heterogeneous (Hetero). ^e^“Validation” shown the large section FISH results in 3 cases obtained from the primary cancer (case #11 and #19) and from the amplified metastasis (#12).

*HER2* amplification according to our selected cut-off (ratio ≥2.0) was observed in a total of 19 of 109 cancers (17.4%). Sixteen of these patients (14.6%) had a high-level amplification (>10 signals) at least in one sample and twelve amplified cases showed large *HER-2* gene signal clusters with a ratio ≥10.0 (11.0%). The relationship between HER2 amplification and tumor phenotype is given in Additional file 1: Table S1. As expected from earlier studies, HER2 amplification was more frequent in intestinal tumors (16%) as compared to diffuse cancers (5%). Significant associations between HER2 amplification and tumor stage, grade or nodal stage were not found in our small set of only 109 cancers. Detailed spot by spot information on each of the 19 patients with at least one amplified tissue sample is given in Figure 2. Ten of these 19 patients showed homogeneous HER2 amplification in all analyzed samples even though the level of amplification varied somewhat from area to area. Three of the nine patients with heterogeneous amplification had heterogeneous findings in the primary tumor that involved high-level amplification (cases #14, #15 and #16). One additional cancer with heterogeneity within the primary tumor (case #17) had a low-level amplification in some but not all spots. Notably, none of these heterogeneously low-level amplified samples had a detectable HER2 overexpression by IHC. Remarkably, there were four amplified tumors cases (#11, #12, #18, #19) that showed high-level amplification in metastases only. Large section validation analysis in three of these cases (#11, #12, and #19) confirmed the high-level amplifications in the metastases of cases #11 and #12, but found case #19 non-amplified. Heterogeneity within the same metastasis, i.e. spots with low-level amplification and spots without amplification in the same metastasis, were not found.

### HER-2 IHC

Positive HER-2 staining with a strong or intermediate circumferential membrane immunostaining (2+ and 3+) was found in 16 of 109 gastric cancers (14.7%). On a spot-by-spot basis, HER-2 expression was strongly linked to HER-2 amplification (Table 1, p < 0.0001). In general, HER2 IHC suggested more heterogeneity in gastric cancer than FISH. Only 5 patients had a 3+ or 2+ IHC result in all evaluable samples. These 5 patients had also homogeneous HER2 amplification. Four other cancers (cases #2, #3, #5, and #6) with homogeneous HER2 amplification were heterogeneous by IHC because of one or several HER2 IHC negative (0/1+) spots. Within the group of 9 “FISH heterogeneous” cases, heterogeneity was also seen by IHC in 7 cancers, while the other 2 cancers were homogeneously IHC negative. While these 2 cases had only low-level amplification, IHC negativity also included 3 spots with high-level amplification (case #10). Heterogeneity within the same metastasis was found in two cases (#2, #13), which showed a discrepant IHC result in one out of three metastases spots each.

**Table 1:** **Association between HER2 FISH and IHC findings in 1,248 tissue spots obtained from 109 gastric cancers**

HER2 FISH	n	HER2 IHC (%)				p-value
		0	1+	2+	3+	
Amplification	150	7.3	14.0	16.7	62.0	*<0.0001*
No amplification	1098	80.5	17.4	1.6	0.5	

### EGFR FISH

**Figure 3:**
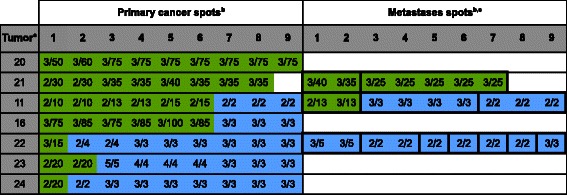
**EGFR FISH findings in primary gastric cancers and their metastases in 7 cancers with EGFR amplification.**^a^“Tumor” indicates the cases number referred to in the text. ^b^The FISH results of individual tissue spots are indicated by integers reflecting the copy numbers of centromere 7/EGFR (i.e. 2/20, black face) in the primary and metastases spots. Green color in the primary/metastases spots area indicates high-level EGFR amplification, and blue color indicates negative IHC findings. ^c^Bold black outline in the “metastases spots” highlights spots that were derived from the same metastasis.

*EGFR* amplification was observed in 7 cancers (6.4%). All amplified cases had high-level amplifications with ≥10 EGFR copies per tumor and showed large EGFR gene signal clusters. Detailed spot by spot information on each of the 7 patients with at least one amplified tissue sample is given in Figure 3. The relationship between EGFR amplification and tumor phenotype is given in Additional file 2: Table S2. Significant associations between EGFR amplification and tumor stage, grade, nodal stage, or histological subtype were not found in our small set of only 109 cancers. Only 2 of 7 amplified cases showed a homogeneous amplification pattern. The other 5 cancers had a heterogeneous situation in the primary tumor. In 3 cancers with metastases, two had an identical result in all analyzable metastases. The remaining case (#11) had one metastasis with high-level amplification and two metastases with normal FISH findings. Heterogeneity of *EGFR* amplification within the same metastasis was not observed.

### *CCND1* FISH

**Figure 4:**
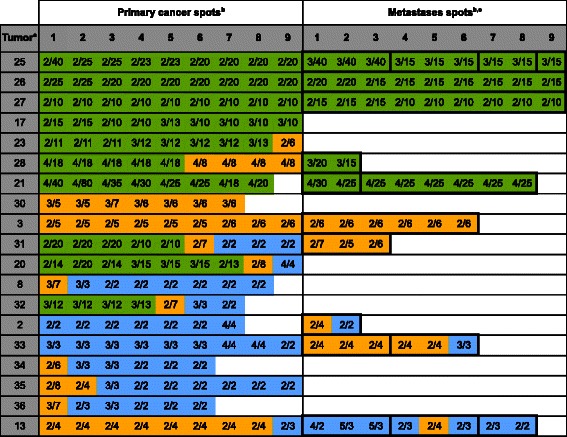
**CCND1 FISH findings in primary gastric cancers and their metastases in 19 cancers with CCND1 amplification.**^a^“Tumor” indicates the cases number referred to in the text. ^b^The FISH results of individual tissue spots are indicated by integers reflecting the copy numbers of centromere 11/CCND1 (i.e. 2/20, black face) in the primary and metastases spots. Green color in the primary/metastases spots area indicates high-level CCND1 amplification, and blue color indicates negative IHC findings. ^c^Bold black outline in the “metastases spots” highlights spots that were derived from the same metastasis.

*CCND1* amplification was observed in 19 of 109 gastric cancers (17.4%). Ten amplified cases showed high-level gene amplification (≥10 *CCND1* signals per cancer cell), including 5 cancers (#21, #25, #26, #28, #31) with large *CCND1* gene signal clusters. There was no obvious association with tumor phenotype (Additional file 2: Table S2). Detailed information on amplified cases is given in Figure 4. Nine of 17 cancers considered amplified in the primary cancer showed homogeneous amplification. This rate was even higher for cancers with high-level amplification (7 of 9). In contrast, only 3 of 10 cancers with a heterogeneous amplification pattern in the primary cancer had spots with high-level amplification. There were two cases with amplification only seen in the nodal metastases (cases #2 and #33). These tumors had a borderline finding in the metastases with 2 centromeres and 4 CCND1 signals per tumor cell. Such borderline findings were also the reason for heterogeneity within the same metastasis that was observed in 3 cases (#2, #33, #13).

### MYC FISH

**Figure 5:**
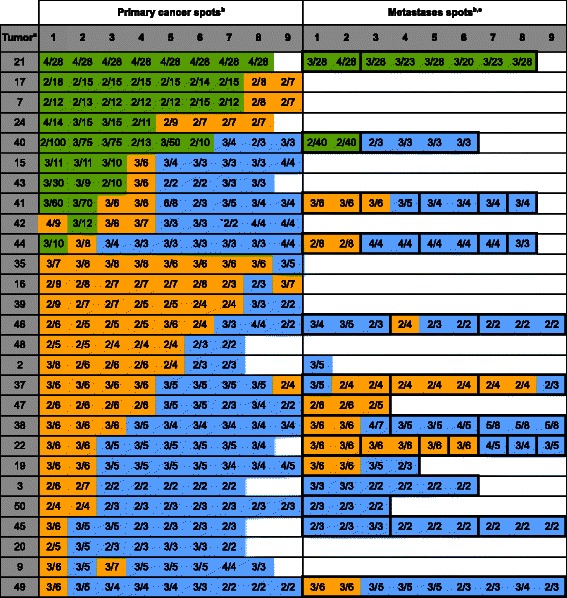
**MYC FISH findings in primary gastric cancers and their metastases in 27 cancers with MYC amplification.**^a^“Tumor” indicates the cases number referred to in the text. ^b^The FISH results of individual tissue spots are indicated by integers reflecting the copy numbers of centromere 8/MYC (i.e. 2/20, black face) in the primary and metastases spots. Green color in the primary/metastases spots area indicates high-level MYC amplification, and blue color indicates negative IHC findings. ^c^Bold black outline in the “metastases spots” highlights spots that were derived from the same metastasis.

*MYC* amplification was observed in 27 of 109 gastric cancers (24.8%). Only 10 (37%) amplified cases showed high-level gene amplification (≥10 MYC signals per cancer cell), including 4 cancers (#21, #40, #41, #43) with large *MYC* gene signal clusters. MYC amplification was significantly linked to intestinal tumors (p = 0.0333). There was no obvious association with tumor phenotype in our set of 109 cancers (Figure 5). Detailed information on amplified cases is given in Figure 4. MYC amplification was typically heterogeneous (23/27 amplified cases, 85%). Presence of heterogeneity was most prevalent in cases with low-level amplification: heterogeneous amplification was found in all (100%) 17 cases with low-level amplification, but only in 6 of 10 (60%) cases with high-level amplification. One of these cancers (#40) had one high-level amplified metastasis and one non-amplified metastasis. The 17 cases with heterogeneous low-level amplification included 8 tumors that had only borderline amplification findings (2–3 centromere and 4–6 MYC signals). Heterogeneity within the same metastasis was observed in 7 cases, but was always caused by borderline findings.

### Co-amplification patterns

**Figure 6:**
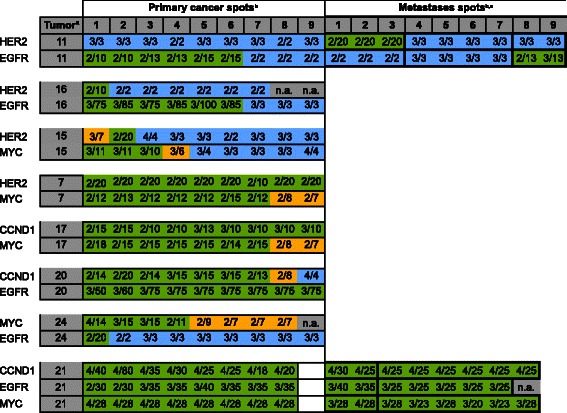
**Comparison of the FISH finding in 8 tumors showing high-level co-amplification of at least 2 of the 4 analyzed genes.**^a^“Tumor” indicates the cases number referred to in the text. ^b^Green color in the primary/metastases spots area indicates high-level amplification, orange color indicates low-level amplification, and blue color indicates negative findings. ^c^Bold black outline in the “metastases spots” highlights spots that were derived from the same metastasis.

Unequivocal co-amplifications involving high-level amplification of at least two of the four genes were found in 8 tumors (Figure 6), including co-amplification of HER2 and EGFR or MYC in 2 cases each, and co-amplifications of EGFR/CCND1, EGFR MYC, CCND1/MYC, and EGFR/MYC/CCND1 in one case each. One case (#11) showed amplification of HER2 and EGFR in different metastases, and had no HER2/EGFR co-amplified primary cancer spots.

## Discussion

Diagnostic accuracy of a molecular assay may be limited if the analyzed biomarker is only present in a fraction of a tumor. Absence of a drug target structure in a cancer subpopulation of a patient tested “positive” for a specific drug target may cause drug resistance after outgrowth of the target-negative population under therapy. Considering its importance, the number of studies systematically analyzing target heterogeneity in cancer is relatively small. Even studies addressing the issue of tumor heterogeneity often limit themselves to the analysis of one slide/block per tumor/patient. However, one tissue section may not completely represent the biology of a large cancer. “Heterogeneity TMAs” were thus manufactured by our group as new tools for studying molecular cancer heterogeneity [18]. A TMA analysis of one sample each from nine different cancer areas obtained from up to nine different blocks distributed across the entire tumor enables a comprehensive three-dimensional high-throughput analysis of molecular features in large series of tumors. The heterogeneity TMA concept introduced here differs markedly from previous attempts to increase the representation of prostate cancer in TMAs by sampling multiple cores from just one tumor block [19,20]. The data of our study demonstrate that heterogeneity of the analyzed amplification occurs in relevant fractions of gastric cancers. Finding substantial off/on heterogeneity for four of four analyzed genes suggests that molecular heterogeneity is rather a rule than an exception for biomarkers in gastric cancer. This clearly limits the potential of treatment decisions based on the amplification status determined on one single clinical cancer biopsy.

HER2 was found amplified in 19 of 109 (17.4%) cancers, including 16 cancers with 3+ positivity by IHC at least in one TMA spot. These data fit well with earlier studies reporting HER2 amplification in 10-27% [21-25] and HER2 overexpression in 5-53% [24-28]. HER2 was the gene with highest homogeneity of amplification in this study. Ten of 12 cancers with large *HER-2* gene signal clusters found in the primary cancer had homogeneous amplification in the entire primary tumor and in all corresponding metastases. This is also consistent with an earlier observation by our group where we found homogeneity of HER2 amplification in 8 of 8 amplified cancers using a different approach. In contrast to our current study, where we systematically sampled all cancers across their entire tumor mass, we had earlier analyzed only one 0.6 mm core per patient and validated the HER2 status on all available tissue blocks of “positive” patients by large section analysis [24]. Our earlier study thus suffered from the potential drawback that the initial screening would enrich for tumors with large HER2 positive areas, and that cancers with HER2 negative status on one 0.6 mm spot were not validated to exclude small HER2 positive cancer areas. The nearly identical findings by two very different but rather systematic approaches corroborate the assumption that the HER2 status is fairly homogeneous in gastric cancer. Clinically, this notion is also supported by the obvious success of trastuzumab therapy in the treatment of HER2 positive gastric cancer, even though HER2 positivity is usually determined on one small piece of tissue only. Other authors had recently reported heterogeneous HER2 findings in 23-79% of HER2 overexpressing or amplified gastric cancers [29-33]. This includes two studies describing HER2 positive nodal metastases in case of HER2 negative primary cancers [26,32]. It is noteworthy, that four such cases were also found in our series. We attribute this phenomenon to small undetected HER2 amplified sub-clones in the primary cancer, as the entire tumor mass cannot be paraffin embedded, retained, and molecularly analyzed in case of large cancers. The high number of such cases raises the question whether a positive HER2 status could indeed facilitate metastasis in gastric cancer. It is further of note, that most metastases were HER2 negative – as the primary cancer – in these patients. This potentially limits the clinical relevance of these HER2 positive metastases.

The interrelation of FISH and IHC data highlights important biological and technical issues related to HER2 in gastric cancer. Technically it is of note, that HER2 IHC results can be influenced by pre-analytical factors such as fixation [13]. Too short fixation results in ethanol exposure of unfixed tissue during tissue processing (dehydration) and can lead to false positive results [34,35]. Prolonged formalin fixation can result in reduced sensitivity of HER2 IHC [36,37]. Biologically, there may be two distinctively different groups of “HER2 positive gastric cancers”. First, there is a fraction of cancers with a massive increase of HER2 signals, which is caused by an intrachromosomal amplification of small stretches of DNA resulting in a clustered arrangement of HER2 FISH signals. These cases have a very high protein overexpression that is mostly detectable by IHC irrespective of some variability in tissue fixation. These cancers often have a homogeneous distribution of amplification and perhaps there is a preference of amplified cancer cells to metastasize. We consider the few samples with negative HER2 IHC in homogeneously amplified cancers as IHC artifacts. Second, there is another group of cancer with a low-level increase of HER2 genes obviously resulting in lower levels of protein overexpression challenging the potential of IHC for reliable detection in a clinical setting. The high rate of discordant IHC/FISH results in this group is thus not surprising. Most of these “amplifications” involve longer stretches of DNA and result from duplications and other rearrangements leading to a more even – non-clustered – arrangement of HER2 signals. Despite attempts to acknowledge the borderline nature of some of these findings by introducing a “borderline” group in the ASCO guidelines for HER2 measurement in breast cancer [38], it is still unclear to what extent these tumors respond to anti-HER2 therapy.

EGFR was found amplified in 7 of 109 cancers (6.4%) in at least one TMA spot. These data fit very well with earlier studies reporting EGFR amplification in 4.9-8.6% by southern blotting [39,40], 7.7-13% by array-based copy number assays [41,42], and 2–6.9% by FISH in larger studies [29]. Higher amplification rates (17-20%) were only reported from small FISH studies analyzing 70–80 cancers [43]. We did not find statistically significant associations between the EGFR amplification status and gastric cancer phenotype in our study, which is at least to a large extent expected due to the small number of amplified cancers. Some earlier studies analyzing 82–511 gastric cancers linked EGFR amplification to advanced and undifferentiated cancers [44] and poor prognosis [45]. EGFR amplification may be of relevance to gastric cancer as a multitude of drugs exists that target EGFR. Four clinical trials had recently not found a survival benefit of gastric cancers treated by standard chemotherapy plus anti-EGFR drugs [46-49], and a retrospective EGFR analysis did not reveal significant associations between the immunohistochemical EGFR expression levels and therapy response or patient survival [48]. However, the vast majority of cancers showed low-level staining according to the various different scoring criteria used in these studies. Given the rarity of high-level EGFR amplification in gastric cancers, a benefit of amplified patients from cetuximab could have potentially been missed. In a recent preclinical study, 4 of 20 gastric cancer xenografts responded favorably to the anti-EGFR antibody cetuximab, two of which had EGFR amplification [50]. A potential benefit of EGFR amplified gastric cancers to anti-EGFR therapies deserves further evaluation in which, however, the frequent heterogeneity of amplification – 5/7 EGFR amplifications were heterogeneous in our patients - will have to be factored in. A randomized phase III trial investigating the addition of nimotuzumab (a monoclonal antibody to EGF), to irinotecan as second line treatment of EGFR overexpressing gastric cancers is now ongoing (ENRICH, ClinicalTrials.gov identifier NCT01813253). Hopefully, the selection of EGFR-positive cancers will enrich for patients who will benefit from anti EGFR therapy.

CCND1 was found amplified in 19 of 109 (17.4%) gastric cancers, including 10 (9.2%) cancers with high-level amplification. This is again in the range of previous studies reporting CCND1 amplification in 17% by southern blot [51], 10% by array-CGH [42] and in 3-10% by FISH [29,52]. CCND1 showed almost the same level of homogeneity of amplification in this study as HER2, with 9 of 19 amplified cases showing amplification of all spots from the primary and metastatic sites. That seven of 10 cases with high-level amplification had a homogeneous FISH result suggests that CCND1 amplification might often represent an early event during tumor development. This is also supported by the fact that we did not find an association between CCND1 amplification and pathological tumor features or histological subtype, which is in concordance with earlier studies on CCND1 amplification or expression in gastric cancer [53]. Of note, Kim et al. [54] reported an association between alcohol consumption and CCND1 amplification in gastric cancer patients, suggesting a specific environmental factor for cancer initiation. Amplification of CCND1 may have potential clinical impact in gastric cancer for two reasons. First, CCND1 amplification has been linked to resistance to the EGFR inhibitor gefitinib in experimental models of head & neck cancer [55], raising the possibility that it might also negatively impact the efficacy of potential anti-EGFR therapy in gastric cancer. Second, several compounds against the CCND1 associated cyclin-dependent kinase CDK4 are currently under investigation in clinical phase I-II trials in multiple cancer types, including an open-label phase I study on advanced solid cancers (NCT01188252) assessing of the impact of CCND1 amplification and expression on therapy efficacy. It will be interesting to see whether CCND1 amplification will turn out as a predictor of response or resistance to such drugs.

MYC was the most frequently amplified gene in our study, with 27 of 109 (24.8%) cancers showing MYC amplification in at least one tumor spot. These numbers are in the upper range of results reported from previous studies finding MYC amplification in 4-26% by southern blot [56-58], 4-30% by array-based or conventional comparative genomic hybridization analysis [42], and 1.3-7.9% by FISH [29,59]. Markedly, higher rates was only reported from two small studies finding MYC amplification in 3 of 7 gastric cancers by FISH [60] and in 17 of 33 tumors using less quantitative PCR for CCND1 amplification analysis [61]. The comparatively high amplification rate found by FISH analysis in our study might have two reasons. First, it is obvious that the analysis of multiple tissue spots from different tumor areas has a higher likelihood to detect amplification in a heterogeneous cancer as if only one sample is analyzed. In fact, MYC amplification showed the highest degree of heterogeneity among the four genes analyzed in our study. Second, most amplifications (i.e., 17 of 27, 63%) were low-level with borderline ratios resulting from 4–6 gene copies and 2–3 centromere copies. Such findings are prone to inter-observer variations even by FISH analysis and it is likely, that the cut-off level of 2.0 for definition of amplification is at least reached once if multiple counts of multiple areas are executed. This is also reflected in our data, since all 17 cases with low-level MYC amplification identified in our study were considered heterogeneous only because of minute copy number variations. It is likely that such low-level amplification is often not MYC specific, but may result from gross chromosomal gains of 8q that belong to the most frequent alterations in gastric cancers [62]. A high fraction of low-level MYC gains in gastric cancers is also confirmed by Suzuki et al. who reported a relative gain (ratio 1.1-1.9) in all of 21 gastric cancers analyzed by MYC-FISH [63]. In contrast, high-level amplification, as indicated by presence of large signal clusters containing more than 10 MYC copies, were found only in 9% of our gastric cancers. All but one of these cancers had areas with and without clear-cut high level amplification or even areas with normal MYC copy numbers, suggesting that high-level MYC amplification occurs at later stages of gastric cancer progression. Finding a significantly higher rate of MYC amplification in intestinal as compared to diffuse cancers suggests a role of MYC for progression of intestinal cancers.

Analyzing four different genes in the same set of 109 cancers allowed us to draw conclusions on the co-amplification patterns of these genes. While on a patient basis, co-amplification of all these genes occurred at frequencies that are compatible with incidental co-alterations, there was one case that showed mutual exclusive amplification HER2 and EGFR in the different tissue spots in the primary tumor and in two metastases. Since HER2 and EGFR receptors activate the same signaling cascades, the finding of mutual exclusive amplification indicates that alteration of either gene is sufficient for gastric cancer cells, and that different subclones of the tumor may have developed HER2 and EGFR amplification independently from each other. Similar conclusion were made by Deng et al. [41] who found copy number alterations of HER2 and EGFR to be restricted to different subsets of gastric cancers. Finding both alterations in different subclones and metastases of the same cancer, however, might provide an explanation for failure of anti-Her2 therapy in some cases. Dual-specific HER2 and EGFR inhibitors like lapatinib may be more effective than single agents in such cases, but may require analysis of multiple tumor areas.

The results of our study underline the importance of taking multiple (6–8) biopsies from different tumor areas for diagnostic purposes as suggested in the guidelines for endoscopic biopsy for evaluation of predictive parameters [64]. This might be particularly important when a highly heterogeneous target gene is analyzed. The example of MYC amplification shows that only small parts of a cancer may harbor such alterations, and that MYC amplification may be present only in as little as two of the nine analyzed cancer regions. It cannot be excluded, however, that more cases with MYC amplification would have been identified if more than 9 cancer spots were analyzed. Of note, findings obtained from small TMA cores (0.6 mm) are not fully interchangeable with clinical biopsies, which typically include a larger amount of tissue. Nevertheless, in case of analyzing intratumoral heterogeneity, the size of the analyzed tissue sample may be less important than sampling from different areas. From a practical point of view, it does not seem to be possible to fully avoid a sampling bias unless the entire tumor has been removed and becomes available for molecular analyses. However, the comparatively low degree of HER2 heterogeneity suggests that currently recommended endoscopic sampling strategies with 6–8 biopsies makes it unlikely that this alteration will be missed when present.

In summary, the results of our study demonstrate intra-tumor heterogeneity in 50-80% of the analyzed primary cancers for the 4 analyzed genes. Although “heterogeneity” was often caused by minute copy number changes resulting in a switch of the diagnostic criteria from “amplified” to “non-amplified” without obvious biological differences, there was also substantial heterogeneity resulting from parallel existence of high-level amplified subclones and those with normal copy numbers. Such “on-off” heterogeneity was frequently found for MYC and EGFR, but rarely for HER2. Tumors harboring multiple subclones with alterations of different therapy target genes are rare but represent an existing category which may open new therapy options based on refined molecular analyses of multiple tumor areas optimally also including metastases.

## Conclusions

Heterogeneity is common for the biomarkers HER2, CCND1, EGFR and MYC in gastric cancer and may therefore limit treatment decisions based on the analysis of a single clinical biopsy.

## Additional files

Additional file 1: Table S1. Association between HER2 alterations and gastric phenotype.
Additional file 2: Table S2. Association between amplification of EGFR, CCND1, and MYC and gastric cancer phenotype.
